# Oncologic safety of sentinel lymph node–based nodal de-escalation in apparent uterine-confined endometrial cancer: a multicenter international cohort study

**DOI:** 10.3389/fonc.2026.1810138

**Published:** 2026-09-03

**Authors:** Alessandro Buda, Ilaria Capasso, Jessica Mauro, Emanuele Perrone, Michael Mueller, Robert Fruscio, Valentina Bruno, Sara Imboden, Tommaso Grassi, Virginia Garcia-Pineda, Elisa Tripodi, Salih Taskin, Stefano Restaino, Diego Raimondo, Vito Andrea Capozzi, Andrea Papadia, Franziska Siegenthaler, Jvan Casarin, Giuseppe Cucinella, Enrico Vizza, Ignacio Zapardiel, Giuseppe Vizzielli, Cagatay Taskiran, Francesco Fanfani

**Affiliations:** 1 Division of Gynecologic Oncology, Michele and Pietro Ferrero Hospital, Verduno, Italy; 2 Dipartimento Scienze della Salute della Donna e del Bambino, Fondazione Policlinico Universitario Agostino Gemelli IRCCS, Rome, Italy; 3 Gynaecological Oncology, Inselspital University Hospital Berne Department of Gynaecology, Bern, Switzerland; 4 Department of Medicine and Surgery, University of Milan-Bicocca, Milan, Italy; 5 Division of Gynecology, Fondazione IRCCS San Gerardo dei Tintori, Monza, Italy; 6 Department of Experimental Clinical Oncology, IRCCS Regina Elena National Cancer Institute, Rome, Italy; 7 Gynecologic Oncology Unit, La Paz University Hospital, Madrid, Spain; 8 Department of Obstetrics and Gynecology, School of Medicine, Ankara University, Ankara, Türkiye; 9 Santa Maria Misericordia Hospital, University of Udine, Udine, Italy; 10 Division of Gynaecology and Human Reproduction Physiopathology, IRCCS Azienda Ospedaliero-Universitaria di Bologna, Bologna, Italy; 11 Department of Obstetrics and Gynecology, University of Parma, Parma, Italy; 12 Department of Gynecology and Obstetrics, Ente Ospedaliero Cantonale, Faculty of Biomedical Sciences, Università della Svizzera Italiana, Lugano, Switzerland; 13 Department of Obstetrics and Gynecology, ‘Filippo Del Ponte’ Hospital, University of Insubria, Varese, Italy; 14 Istituto Nazionale dei Tumori, Fondazione Pascale, Napoli, Italy; 15 Department of Obstetrics and Gynecology, School of Medicine, Koc University, Istanbul, Türkiye; 16 Dipartimento Scienze della Vita e Sanità Pubblica, Università Cattolica del Sacro Cuore Facoltà di Medicina e Chirurgia, Rome, Italy

**Keywords:** endometrial cancer, nodal de-escalation, recurrence, sentinel lymph node, tumor biology

## Abstract

**Background:**

Sentinel lymph node (SLN) mapping has increasingly replaced systematic lymphadenectomy in apparent uterine-confined endometrial cancer (EC). However, concerns persist regarding the risk of recurrence following nodal surgical de-escalation, particularly in patients with aggressive histologic subtypes. We aimed to evaluate the oncologic safety of SLN-based nodal de-escalation by analyzing recurrence patterns and recurrence-free survival in patients with apparent early-stage EC.

**Methods:**

We conducted a retrospective multi-institutional study including women with apparent uterine-confined EC who underwent primary surgery including SLN mapping, with or without pelvic and/or para-aortic lymphadenectomy. Patients were grouped according to nodal staging strategy: SLN-only, SLN plus pelvic lymphadenectomy (PLND), and SLN plus PLND plus para-aortic lymphadenectomy (PALND). The primary endpoints were progression-free survival (PFS) and patterns of recurrence.

**Results:**

We included 2123 patients from 15 centers in six countries. SLN-only staging was performed in 1,341 patients (63.2%), SLN+PLND in 483 (22.8%), and SLN+PLND+PALND in 299 (14.1%). With a median follow-up of 44.9 months (IQR 19.1–73.4), 121 recurrences were observed (5.6%). No statistically significant differences in PFS were observed among nodal staging groups. On multivariable Cox analysis, SLN-only staging was not associated with inferior PFS compared with SLN+PLND (HR 1.12, p=0.61), and the addition of PALND did not confer a significant benefit. Endometrioid high-grade histology, non-endometrioid high-risk histotypes, deep myometrial invasion, and lymphovascular space invasion were independently associated with recurrence. Isolated nodal relapse was uncommon (14.9%) and similarly distributed across groups. Exploratory molecular analysis did not show statistically significant differences in PFS across molecular subgroups, although expected survival trends were observed.

**Conclusions:**

In apparent uterine-confined EC, no significant differences in recurrence or nodal relapse were observed across nodal staging strategies. These findings support the use of SLN mapping as an adequate staging approach within a biology-driven framework, although they should be interpreted in light of the retrospective design.

## Highlights

- No significant differences in recurrence or nodal relapse were observed across nodal staging strategies in apparent uterine-confined endometrial cancer.
- The extent of nodal surgery was not associated with differences in recurrence-free survival or recurrence patterns.
- Recurrence risk appears to be largely influenced by tumor biology and histologic risk factors rather than by the extent of nodal surgery.
- These findings support the use of sentinel lymph node mapping within a biology-driven approach to surgical staging, while acknowledging the limitations of the retrospective design.

## Introduction

Endometrial cancer (EC) is the most common gynecologic malignancy in high-income countries, with a steadily increasing incidence worldwide. Most patients are diagnosed with disease that is clinically confined to the uterus, and primary surgery represents the cornerstone of treatment. In this context, accurate surgical staging is essential for prognostic stratification and for guiding adjuvant treatment decisions (1). Two randomized trials failed to demonstrate a survival benefit associated with systematic lymphadenectomy in early-stage disease, while consistently reporting increased operative time, blood loss, and long-term morbidity, particularly lymphedema (2, 3). These findings prompted a paradigm shift toward less invasive approaches to nodal assessment. Sentinel lymph node (SLN) mapping subsequently emerged as a reliable alternative to systematic lymphadenectomy. Large prospective and retrospective series have demonstrated high detection rates, sensitivity, and negative predictive value for nodal metastases, particularly when standardized algorithms, cervical injection techniques, and ultrastaging protocols are applied (4, 5). As a result, SLN mapping has been incorporated into international guidelines as an acceptable and often preferred staging strategy for apparent uterine-confined EC. The recently updated ESGO–ESTRO–ESP guidelines (6) further emphasize a risk-adapted approach to surgical staging, recognizing SLN mapping as the standard nodal assessment technique in most patients with apparent early-stage disease, while discouraging routine systematic lymphadenectomy in the absence of specific risk factors. In parallel, increasing attention has been directed toward tumor biology, histologic subtype, and molecular classification as key determinants of prognosis and treatment selection (7). Despite the widespread adoption of SLN mapping, some concerns remain regarding the long-term oncologic safety of nodal de-escalation. In particular, the potential risk of nodal recurrence following SLN-only staging is still debated, especially in patients with high-risk histologic subtypes characterized by aggressive behavior and early lymphovascular dissemination (8). While several studies have evaluated survival outcomes after SLN mapping, data specifically addressing nodal relapse as a distinct failure pattern remain limited. Non-endometrioid carcinomas and high-grade endometrioid tumors exhibit distinct patterns of spread and impaired prognosis compared with low-risk tumors. Whether these biologic differences translate into differential risks of nodal recurrence following surgical de-escalation remains poorly understood in large, real-world cohorts. The primary objective of this international collaborative study was to evaluate the oncologic safety of nodal de-escalation in apparent uterine-confined EC, with a specific focus on progression-free survival and patterns of recurrence, including isolated nodal relapse. A secondary objective was to evaluate histotype- and molecular-specific risks of nodal upstaging and recurrence, to better characterize the biological determinants of disease spread and relapse.

## Materials and methods

### Study design and population

This retrospective cohort study included consecutive women with endometrial cancer treated at 15 tertiary referral centers from six countries. Institutional databases were queried to identify patients who underwent primary surgical treatment between August 2012 and February 2024. The study was approved by the institutional review board of the promoting institution at Michele and Pietro Ferrero Hospital (IRB approval number 570/CE 4), and local ethical approval was obtained from all participating centers according to local regulations. All patients provided informed consent for the use of their clinical data. Clinicopathologic data were extracted from a centralized, anonymized database and reviewed for completeness and consistency prior to final analysis. Eligible patients had apparent uterine-confined endometrial cancer based on preoperative biopsy. Preoperative imaging included expert transvaginal ultrasound and/or either computed tomography, or positron emission tomography/computed tomography (PET/CT). Patients with evidence of extra-uterine disease at diagnosis, those treated with neoadjuvant therapy, previous pelvic or abdominal irradiation (including brachytherapy), were excluded.

### Surgical staging strategies

All patients underwent surgical staging including hysterectomy, bilateral salpingo-oophorectomy, and SLN mapping, with or without additional pelvic and/or para-aortic lymphadenectomy. Given the long study period (2006–2021), SLN mapping techniques evolved over time according to institutional practice. Until approximately 2014, SLN biopsy was predominantly performed using blue dye alone or blue dye in combination with technetium-99m (Tc99m) radiocolloid, as previously reported (9). Thereafter, indocyanine green (ICG) was progressively introduced and became the preferred tracer in several participating centers. Although minor variations in tracer selection existed across institutions, all centers were tertiary referral hospitals performing SLN mapping according to internationally accepted principles and standardized surgical algorithms. Mapping was considered successful when at least one SLN was identified. Pelvic and/or para-aortic lymphadenectomy was performed according to intraoperative findings and perceived risk factors until publication of the FIRES trial (4), which demonstrated the feasibility and high diagnostic accuracy of SLN mapping in stage I endometrial cancer. Thereafter, nodal staging followed the Memorial Sloan Kettering Cancer Center SLN algorithm for SLN mapping (10). All SLNs underwent ultrastaging according to WHO guidelines (11, 12). SLN status was classified as negative, isolated tumor cells (≤0.2 mm or <200 cells), micrometastases (>0.2 mm to ≤2.0 mm or >200 cells), or macrometastases (>2.0 mm).

According to the extent of nodal staging, patients were classified into three groups: 1) SLN-only; 2) SLN plus pelvic lymphadenectomy (PLND); 3) SLN plus PLND plus para-aortic lymphadenectomy (PALND).

### Histotype classification

Tumors were classified into four clinically meaningful categories: endometrioid low-grade (G1–2), endometrioid high-grade (G3), non-endometrioid high-risk histotypes (serous, clear cell, undifferentiated, and high-grade mixed carcinomas), and carcinosarcoma.

### Molecular analysis

Molecular analyses were restricted to patients with complete molecular classification according to the full algorithm (including POLE, MMR, and p53 status). Molecular analysis was performed on preoperative biopsy or final surgical specimens. Tumors were classified as: POLE-mutated, based on pathogenic exonuclease domain mutations; MMR-deficient (MMRd), defined by loss of expression of one or more MMR proteins (PMS2, MLH1, MSH6, MSH2); p53-abnormal, defined by aberrant p53 immunostaining, considered a reliable surrogate of TP53 mutation; Nonspecific molecular profile (NSMP), triple negative.

Testing methodologies included next-generation sequencing and Sanger sequencing. In selected cases, TP53 status was confirmed by extended sequencing. In accordance with ESGO recommendations, POLE testing was not routinely performed in low- and intermediate-risk tumors (6).

### Statistical analysis

The primary endpoints were progression-free survival (PFS) and patterns of recurrence (pelvic, extra-pelvic/distant, isolated nodal). Multi-site recurrences were classified according to all involved sites. Baseline characteristics were compared across nodal staging strategies. PFS was estimated using the Kaplan–Meier method and compared using Cox proportional hazards models. Multivariate models were adjusted for histotype, depth of myometrial invasion (>50%), lymphovascular space invasion (LVSI), and cervical stromal involvement. Logistic regression was used to assess predictors of nodal recurrence and nodal upstaging to stage IIIC. Robust standard errors accounted for center clustering. Statistical significance was set at p<0.05. Analyses were performed using SPSS software (*IBM Corp., Armonk, NY, USA*). Complete-case analysis was applied. Data handling followed previously described SLYMEC methodology (13, 14).

## Results

### Patient characteristics

A total of 2123 patients with uterine-confined EC were included. SLN-only staging was performed in 1,341 patients (63.2%), SLN+PLND in 483 (22.8%), and SLN+PLND+PALND in 299 (14.1%). The median number of SLNs removed was 2 (IQR, 2–4). SLN mapping was successful in 2,060 of 2,123 patients (97.0%). Bilateral mapping was achieved in 1,667 patients (78.5%), whereas unilateral mapping occurred in 393 (18.5%). No SLN migration was observed in 63 patients (3.0%). Overall, 319 patients (15%) were shown to have positive lymph nodes at final pathology. Only 19 (0.9%) women had positive non-SLN with negative SLNs. Patients undergoing more extensive nodal staging more frequently presented adverse clinicopathologic features, including high-grade histology, deep myometrial invasion, and LVSI. Baseline demographic, surgical, and clinicopathologic characteristics according to nodal staging strategy are summarized in **Table 1**.

**Table 1:** Baseline clinicopathologic characteristics of the overall study population (N = 2123).

Variable	Overall
Age (years), median (IQR)	63 (55–70)
BMI (kg/m²), median (IQR)	28.3 (24.3–33.8)
Surgical approach, n (%)	
MIS traditional	1,509 (71.1)
MIS robotic-assisted	494 (23.3)
Open approach	120 (5.6)
Histology, n (%)	
Endometrioid low-grade (G1–2)	1,152 (54.3)
Endometrioid high-grade (G3)	456 (21.5)
Non-endometrioid high-risk	515 (24.2)
Myometrial invasion ≥50%, n (%)	768 (36.2)
LVSI present, n (%)	571 (26.9)
Cervical stromal involvement, n (%)	206 (9.7)
Nodal staging strategy, n (%)	
SLN-only	1,341 (63.2)
SLN + PLND	483 (22.8)
SLN + PLND + PALND	299 (14.1)
N. of SLN's removed, median (IQR)	2 (2-4)
Mapping, n (%)	

Legend. N, number; BMI, body mass index; IQR, inter quartile range; Pts, patients; MIS, minimally invasive surgery; LVSI, lymphovascular space invasion; SLN, sentinel lymph node; PLND, pelvic lymphadenectomy; PALND, aortic lymphadenectomy; RT, radiotherapy; CT, chemotherapy; RCT, radiochemotherapy; NSMP, not specific molecular profile.

### Patterns of recurrence

After a median follow-up of 44.9 months (IQR 19.1–73.4), 121 recurrences were observed (5.6%). Nodal involvement occurred in 18 cases (14.9%). The proportion of nodal recurrences did not differ across nodal strategies (SLN-only 13.2%, SLN+PLND 16.1%, SLN+PLND+PALND 16.2%, p value=0.99). Patterns of recurrence according to nodal staging strategy are summarized in **Table 2**. Among patients with isolated nodal recurrence, 83.3% were node-negative at initial staging. Only three patients had SLN metastases at diagnosis including one ITC, and two with macrometastases. No patients with micrometastases developed isolated nodal recurrence Detailed characteristics of patients with isolated nodal recurrence are reported in **Supplementary Table 1**.

**Table 2:** Pattern of recurrence according to nodal staging strategy (N=121).

Site of recurrence	SLN-only	SLN+PLND	SLN+PLND+PALND	Overall	p-value*
Any recurrence	85 (6.3)	24 (5.0)	12 (4.0)	121 (5.6)	0.055
Pelvic	15 (17.6)	3 (12.5)	2 (16.7)	20 (16.5)	N.S.
Extra-pelvic/distant	21 (24.7)	6 (25.0)	5 (41.7)	32 (26.4)	0.760*
Isolated nodal	13 (15.3)	3 (12.5)	2 (16.7)	18 (14.9)	0.586**

Legend. SLN, sentinel lymph node; PLND, pelvic lymphadenectomy; PALND, aortic lymphadenectomy; N.S., not significant. [Note. The p-value refers to comparison across nodal staging groups: * Chi-square test; ** Fisher exact test].

### analyse Histotype-specific s

Nodal upstaging to stage IIIC was significantly higher in non-endometrioid high-risk tumors (19.3%) compared with endometrioid low-grade tumors (6.7%). On multivariate analysis, non-endometrioid histology remained independently associated with nodal upstaging (OR 2.41, p<0.001). Recurrence rates increased progressively across histologic risk categories. Multivariate analyses for nodal upstaging are shown in **Table 3A**.

**Table 3:** Multivariate analyses.

A) Logistic regression analysis for nodal upstaging to FIGO stage IIIC			
Variable	OR	95% CI	p-value
Endometrioid high-grade	1.42	0.91–2.21	0.12
Non-endometrioid high-risk	2.41	1.56–3.72	<0.001
Myometrial invasion ≥50%	1.88	1.22–2.89	0.004
LVSI present	2.67	1.74–4.10	<0.001
B) Cox proportional hazards model for recurrence-free survival			
Variable	OR	95% CI	p-value
SLN-only vs SLN+PLND	1.12	0.73–1.71	0.61
SLN+PLND+PALND vs SLN+PLND	0.98	0.61–1.59	0.94
Endometrioid high-grade	1.83	1.11–3.01	0.017
Non-endometrioid high-risk	2.13	1.33–3.41	0.002
Myometrial invasion ≥50%	1.69	1.12–2.55	0.012
LVSI present	1.94	1.29–2.92	0.001

Legend. SLN, sentinel lymph node; PLND, pelvic lymphadenectomy; PALND, aortic lymphadenectomy; N, number. LVSI, lympho vascular space invasion; CI, Confidence interval.

### Survival analysis

**Figure 1:**
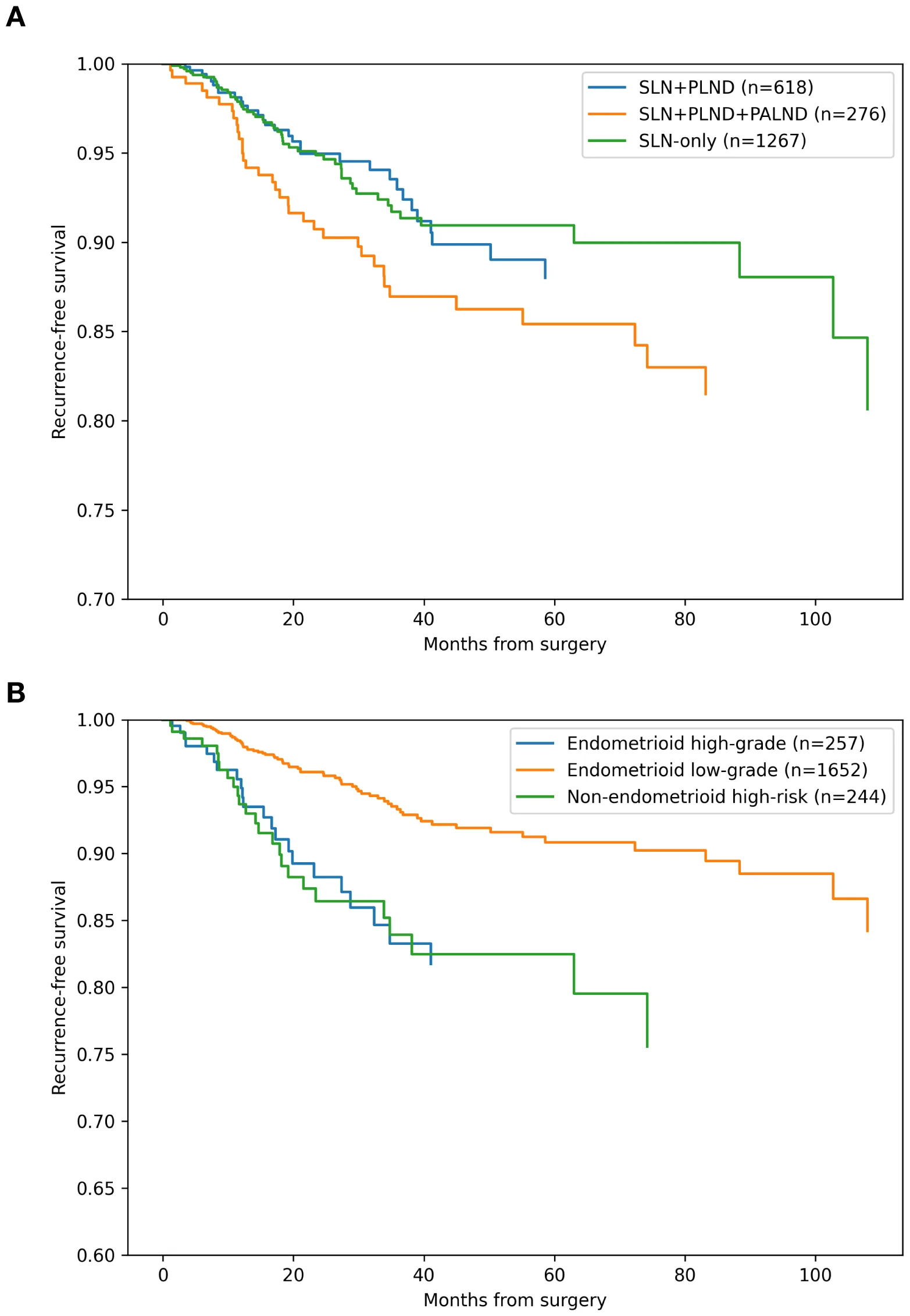
Kaplan–Meier curves of progression-free survival (PFS). **(A)** PFS curves according to nodal staging strategy: sentinel lymph node mapping alone (SLN-only), SLN plus pelvic lymphadenectomy (SLN+PLND), and SLN plus pelvic and para-aortic lymphadenectomy (SLN+PLND+PALND). No significant differences in PFS were observed among the three groups (log-rank p = 0.58). **(B)** PFS curves stratified by histologic subtype. Endometrioid low-grade tumors demonstrated the most favorable outcomes, whereas non-endometrioid high-risk histotypes endometrioid high-grade tumors profile were associated with the poorest PFS (log-rank p < 0.001).

**Figure 2:**
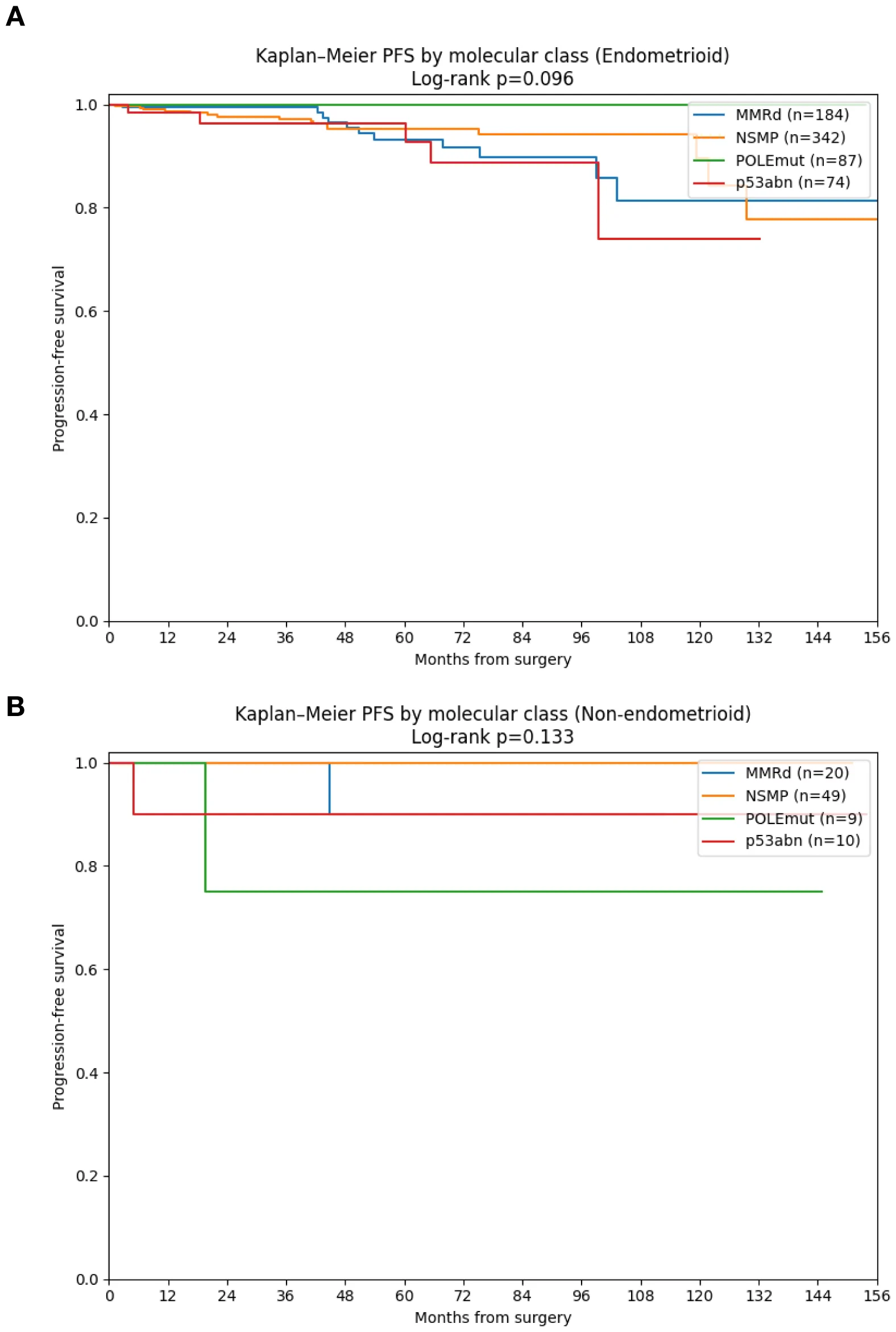
Progression-free survival (PFS) according to histologic subtype restricted to patients with complete molecular classification (n=834). **(A)** Progression-free survival by molecular class stratified by endometrioid carcinomas (log-rank p = 0.096); **(B)** Progression-free survival by molecular class stratified by non-endometrioid carcinomas (log-rank p = 0.133).

On multivariate Cox regression analysis, SLN-only staging was not associated with inferior PFS compared with SLN plus pelvic lymphadenectomy (HR 1.12, p = 0.61) or SLN plus pelvic and para-aortic lymphadenectomy (HR 0.98, p = 0.94). In contrast, endometrioid high-grade tumors (HR 1.83, p = 0.017), non-endometrioid high-risk histotypes (HR 2.13, p = 0.002), deep myometrial invasion (HR 1.69, p = 0.012), and lymphovascular space invasion (HR 1.94, p = 0.001) were independently associated with increased recurrence risk (**Table 3B**). Kaplan–Meier analysis showed no significant differences in PFS according to nodal staging strategy (log-rank p = 0.58; **Figure 1**). Patients undergoing SLN-only staging demonstrated recurrence-free survival comparable to those treated with additional pelvic and/or para-aortic lymphadenectomy. In contrast, PFS differed significantly according to histologic subtype (log-rank p < 0.001; **Figure 2**). Endometrioid low-grade tumors showed the most favorable outcomes, whereas non-endometrioid high-risk histotypes were associated with the poorest PFS, with endometrioid high-grade tumors exhibiting an intermediate risk profile. While nodal staging strategy did not significantly influence PFS, histologic subtype and molecular classification emerged as the major determinants of outcome. To further assess the potential impact of adjuvant treatment, sensitivity analyses were performed in two complementary settings: a high-risk subgroup and patients who did not receive adjuvant therapy. In both analyses, recurrence rates remained comparable across nodal staging strategies (high-risk subgroup: p=0.736; no adjuvant treatment: p=0.161).

### Exploratory molecular analysis

Molecular classification included four groups according to the TCGA/ProMisE framework: POLE-mutated (POLEmut), mismatch repair-deficient (MMRd), p53-abnormal (p53abn), and no specific molecular profile (NSMP). Molecular classification was available for 834 patients with complete data, including POLEmut (n=108), MMRd (n=226), p53abn (n=82), and NSMP (n=418). Recurrence rates differed numerically across molecular subgroups, with POLE-mutated tumors showing the lowest rate of recurrence and p53-abnormal tumors the highest, while MMR-deficient and NSMP tumors demonstrated intermediate outcomes. However, Kaplan–Meier analysis of PFS according to molecular classification did not show statistically significant differences between groups (log-rank p=0.096). No significant interaction between molecular subtype and nodal staging strategy was observed. These analyses should be considered exploratory given the limited number of events and the incomplete availability of molecular data across the entire cohort.

## Discussion

### Summary of main findings

This large international multi-institutional study including 2123 women with apparent uterine-confined endometrial cancer represents, to our knowledge, the largest series reported in this setting. Our findings demonstrate that nodal de-escalation using SLN mapping alone was not associated with differences in recurrence or nodal relapse compared with more extensive nodal staging strategies. Recurrence-free survival did not differ significantly across nodal strategies (log-rank p = 0.58), and SLN-only staging was associated with comparable outcomes compared with SLN plus pelvic lymphadenectomy (HR 1.12; 95% CI 0.73–1.71, p = 0.61). Among relapsed patients, isolated nodal relapse was uncommon, accounting for 18 of 121 recurrences (14.9%), with comparable proportions observed across the SLN-only, SLN+PLND, and SLN+PLND+PALND groups. Importantly, most nodal-only recurrences occurred in patients who were node-negative at initial staging (15/18, 83.3%), suggesting that nodal relapse does not simply reflect residual nodal disease but may represent aggressive tumor biology or alternative dissemination pathways. Tumor-related factors, rather than nodal surgical extent, emerged as the main determinants of recurrence. On multivariate analysis, non-endometrioid high-risk histotypes (HR 2.13, p = 0.002), endometrioid high-grade tumors (HR 1.83, p = 0.017), deep myometrial invasion (HR 1.69, p = 0.012), and lymphovascular space invasion (HR 1.94, p = 0.001) were independently associated with worse recurrence-free survival. Similarly, non-endometrioid high-risk histotypes were strongly associated with nodal upstaging to FIGO stage IIIC (OR 2.41, p < 0.001). Although patients undergoing more extensive nodal staging more frequently received adjuvant chemotherapy or combined chemoradiotherapy, recurrence-free survival did not differ according to nodal strategy, suggesting that differences in outcome are unlikely to be primarily driven by treatment intensity but by intrinsic tumor characteristics.

### Comparison with previous studies

Our findings are consistent with, and extend, prior evidence questioning the therapeutic value of extensive nodal surgery in endometrial cancer. Two randomized trials demonstrated that systematic lymphadenectomy improves nodal staging accuracy but does not translate into improved survival outcomes, while increasing surgical morbidity (2, 3). In selected high-risk settings, a potential therapeutic role of nodal debulking has been hypothesized. Swift et al. (15) evaluated patients with high-grade endometrial cancer and at least one positive lymph node, demonstrating that removal of a higher number of metastatic lymph nodes (>15) was associated with longer recurrence-free survival. Importantly, this study addressed patients with proven nodal disease and does not support routine lymphadenectomy in node-negative patients, as well as does not contradict the safety of SLN-based staging in apparent uterine-confined disease. Most contemporary studies focusing on SLN mapping have reported consistently low rates of nodal recurrence. Similarly, Lee et al. (16), in a large propensity score–matched analysis, reported no difference in progression-free survival between SLN biopsy and systematic lymphadenectomy. Nodal recurrence occurred in 4 patients (1.56%) in the SLN group and in 9 patients (3.5%) in the lymphadenectomy group, with no statistically significant difference between strategies (p=0.130). In an Italian retrospective study including 802 patients with apparent early-stage endometrial cancer, no difference in survival between patients managed with a SLN–based algorithm and those undergoing selective lymphadenectomy (17). Several additional retrospective series have confirmed the oncologic safety of SLN-based algorithms; however, many did not report detailed site-specific recurrence patterns, limiting direct comparison with our analysis specifically focused on nodal failure (18–21).

### Tumor biology, histology, and nodal disease burden

Beyond surgical extent, increasing attention has been directed toward the prognostic relevance on the type of SLN involvement. A recent systematic review and meta-analysis by Bogani et al. (22) confirmed that low-volume SLN metastases are not independently associated with worse survival once tumor-related factors are considered. Similarly, How et al. (23) reported a low rate of nodal recurrence in 472 who underwent either SLN biopsy, or systematic. Authors concluded that SLN mapping may enable more efficient detection of lymph node involvement and help to guide adjuvant therapy, without increasing pelvic sidewall failures. The prognostic relevance of histology observed in our study aligns with prior literature. Bak et al. (24), demonstrated that histological grade retains prognostic significance even among low-risk endometrioid tumors, reporting recurrence rates of approximately 3–5% in low-grade disease compared with rates approaching 10% in higher-grade tumors. Similarly, Güngördük et al. (25) similarly identified tumor grade, depth of myometrial invasion, and lymphovascular space invasion as the main predictors of recurrence in apparent uterine-confined disease, underscoring that relapse may occur despite favorable stage at presentation. In our cohort, recurrence rates followed a clear biologic gradient: 4.4% in endometrioid low-grade tumors, 8.9% in endometrioid high-grade tumors, and 10.1% in non-endometrioid high-risk histotypes (p < 0.001). On multivariate analysis, histologic subtype remained independently associated with recurrence. Recurrence remained comparable across nodal staging strategies. Sensitivity analyses in high-risk patients and in those not receiving adjuvant treatment showed consistent results, suggesting that the findings are unlikely to be primarily driven by differences in adjuvant treatment, although residual confounding cannot be excluded. Importantly, isolated nodal relapse was rare and predominantly occurred in patients without nodal involvement at diagnosis, and no significant differences in recurrence patterns were observed according to the type of SLN metastasis (χ² p value = 0.59; **Supplementary Table 1**).

### Molecular classification and recurrence risk

Molecular profiling has emerged as a critical determinant of outcome. The molecular analysis of the PORTEC-3 trial by León-Castillo et al. (26) demonstrated marked differences in recurrence-free survival across TCGA-like molecular classes, with excellent outcomes in POLE-mutated tumors and the poorest prognosis in p53-abnormal cancers. Moreover, the combined analysis of the PORTEC cohorts by Stelloo et al. (7) demonstrated that integrating molecular features with clinicopathologic factors significantly improves risk stratification and recurrence prediction compared with traditional histopathologic assessment alone. In line with these observations, exploratory molecular analyses showed trends consistent with known biological behaviour. Although differences in progression-free survival across molecular subgroups did not reach statistical significance (log-rank p=0.096), POLE-mutated tumors demonstrated the most favourable outcomes, while p53-abnormal tumors tended to show poorer prognosis. No significant interaction was observed between molecular subtype and nodal staging strategy. However, these findings should be interpreted with caution given the limited number of events, the relatively short follow-up, and the subset of patients with available molecular data. These results are further supported by Mariano et al. (27), who demonstrated that molecular classification retained independent prognostic significance in high-risk endometrial cancer patients uniformly staged with SLN mapping. A recent systematic review by Bassi et al. (28) also suggested that the prognostic impact of low-volume nodal metastases varies according to molecular subtype. Specifically, low-volume nodal disease appeared to have minimal prognostic impact in favorable molecular classes, such as POLE-mutated tumors, while potentially carrying greater relevance in p53-abnormal cancers. These findings further highlight that nodal disease burden alone does not uniformly translate into recurrence risk and must be interpreted within the broader context of tumor biology. Histotype emerged as a major determinant of both nodal upstaging and recurrence. Non-endometrioid high-risk tumors demonstrated nodal upstaging rates approaching 20% and recurrence rates exceeding 10%, compared with substantially lower rates in endometrioid low-grade tumors. Endometrioid high-grade carcinomas showed an intermediate risk profile, underscoring the heterogeneity within endometrioid disease and supporting grade-based stratification.

### Strengths, limitations, and clinical implications

The strengths of this study include the large sample size, the international multicenter design involving tertiary centers, and the detailed assessment of recurrence patterns, with a specific focus on nodal relapse. The integration of histologic and molecular data allows a biologically informed interpretation of recurrence risk beyond anatomic staging alone. In particular, the characterization of nodal-only recurrences provides clinically relevant insight, showing that most nodal relapses occurred in patients who were node-negative at final pathology. Limitations include the retrospective design and the inherent potential for selection bias in the choice of nodal staging strategy, reflecting real-world, risk-adapted surgical practice. Accordingly, these findings should be interpreted with caution. While no significant differences in survival were observed, the results support the oncologic safety of SLN-only staging within these limitations rather than establishing equivalence between strategies.

The relatively short median follow-up may underestimate late recurrences in some subgroups, and the low number of nodal events limited the power of subgroup analyses focused on nodal relapse. In addition, molecular data were available only in a subset of patients (834/2123), limiting the ability to draw definitive conclusions regarding molecular-guided surgical decision-making and precluding formal interaction analyses between molecular subtype, adjuvant therapy, and nodal strategy. These molecular findings should be interpreted as exploratory and hypothesis-generating, given the limited availability of complete molecular data and the potential for selection bias.

### Ongoing studies and future perspectives

Several recent and ongoing studies are specifically investigating the integration of molecular profiling with SLN assessment in endometrial cancer, with the aim of refining surgical staging within a biology-driven framework. The SENECA study (29) demonstrated significant heterogeneity in SLN involvement across molecular subtypes, reporting the highest nodal metastasis rates in p53-abnormal tumors and substantially lower rates in POLE-mutated and NSMP cancers. These findings further support the concept that nodal dissemination risk is closely linked to underlying tumor biology rather than to clinicopathologic factors alone. Similarly, Siegenthaler et al. (30) showed that SLN mapping retains independent prognostic value when interpreted within molecular subgroups, reinforcing the role of SLN assessment as part of an integrated biologic staging approach rather than as a purely anatomic procedure. Prospective and randomized trials are now actively refining SLN-based staging algorithms through molecular integration. The SELECT study and the randomized ALICE trial (31, 32) are evaluating whether molecular risk stratification can guide the extent of nodal staging and adjuvant treatment, with the goal of safely de-escalating surgical morbidity in selected patient subgroups. The ENDO-3 trial is further addressing the role of SLN mapping by directly testing whether omission of additional retroperitoneal lymph node dissection can be oncologically safe in apparent early-stage disease (33). Furthermore, the ongoing SENTIMETREP53 trial focuses specifically on patients with p53-mutated endometrial cancer and directly compares SLN mapping with comprehensive lymphadenectomy (34). This study is expected to provide critical evidence on whether even molecularly aggressive tumors truly require extensive nodal dissection or can be safely managed with SLN-based staging. Collectively, these trials are anticipated to clarify the role of molecular classification in guiding nodal assessment and to define which subgroups may safely undergo surgical de-escalation without compromising oncologic outcomes.

### Clinical implications and future directions

Our findings support SLN–based staging as a standard surgical approach in apparent uterine-confined endometrial cancer. In our cohort, recurrence-free survival did not differ according to nodal strategy, while nodal relapse accounted for a minority of failures (14.9%) and predominantly occurred in patients who were node-negative at initial staging (83%). These results reinforce the concept that nodal surgical extent does not appear to independently modify recurrence risk in early-stage disease and that lymph node assessment should be primarily performed with staging purposes within a broader biologic risk framework. In this context, SLN biopsy minimizes surgical morbidity without compromising oncologic safety. Emerging artificial intelligence and machine learning approaches provide a promising framework to integrate clinicopathologic, histologic, and molecular variables for individualized recurrence-risk prediction. As recently reported (35, 36), machine learning models combining guideline-based risk criteria with immune and molecular features may improve prognostic stratification beyond traditional models. Thus, in combination with SLN mapping those tools, further improve identification of patients who would benefit most from tailored adjuvant therapies or intensified surveillance, while supporting safe surgical de-escalation in lower-risk profiles.

## Conclusions

In apparent uterine-confined endometrial cancer, no significant differences in recurrence or nodal relapse were observed across nodal staging strategies. In this large retrospective multicenter cohort, although nodal involvement was more frequent in biologically aggressive tumors, the extent of nodal dissection was not associated with improved recurrence outcomes. Recurrence risk appears to be largely influenced by tumor biology, including histology, grade, and molecular subtype, rather than by the extent of nodal surgery. These findings support the use of SLN mapping as an adequate staging approach within a personalized, biology-driven management strategy, while acknowledging the limitations inherent to the retrospective design and potential selection bias. Future integration of molecular profiling with advanced risk prediction tools, including machine learning–based models, may further refine individualized treatment and surveillance pathways in endometrial cancer.

## Data Availability

The raw data supporting the conclusions of this article will be made available by the authors, without undue reservation.

## Supplementary material

Supplementary Figure 1 Analysis restricted to patients with complete molecular classification (n=834). Progression-free survival by molecular class stratified by endometrioid carcinomas (log-rank p = 0.096).

Supplementary Figure 2 Analysis restricted to patients with complete molecular classification (n=834). Progression-free survival by molecular class stratified by non-endometrioid carcinomas (log-rank p = 0.133).
