# Supplementary material for: Oncologic safety of sentinel lymph node–based nodal de-escalation in apparent uterine-confined endometrial cancer: a multicenter international cohort study
Source: Front Oncol. 2026 Sep 3;16:1810138. doi: 10.3389/fonc.2026.1810138 (PMC13581940; doi:10.3389/fonc.2026.1810138)
Supplement: Supplementary file 1 [file Table1.docx]

**Table S1. Characteristics of patients with isolated nodal recurrence (n = 18)**

| **Variable** | **n (%)** |
| --- | --- |
| **Nodal staging strategy** |  |
| SLN-only | 8 (44.4) |
| SLN + PLND | 6 (33.3) |
| SLN + PLND + PALND | 4 (22.2) |
|  |  |
| **Nodal status at initial staging** |  |
| Negative | 15 (83.3) |
| SLN-positive | 3 (16.7) |
| *ITC* | *1 (5.6)* |
| *MIC* | *0 (0)* |
| *MAC* | *2 (11.1)* |
|  |  |
| **Histology at diagnosis** |  |
| Endometrioid low-grade (G1–2) | 5 (27.8) |
| Endometrioid high-grade (G3) | 6 (33.3) |
| Non-endometrioid high-risk | 6 (33.3) |
| Carcinosarcoma | 1 (5.6) |
|  |  |
| **Myometrial invasion ≥50%** | 11 (61.1) |
| **LVSI present** | 13 (72.2) |
| **Cervical stromal involvement** | 4 (22.2) |
|  |  |
| **Adjuvant treatment** |  |
| None | 2 (11.1) |
| RT alone | 5 (27.8) |
| CT alone | 2 (11.1) |
| RCT | 9 (50.0) |

**Legend.** SLN: sentinel lymph node; LVSI: lympho vascular space invasion; PLND: pelvic lymphadenectomy; PALND: aortic lymphadenectomy; ITC: isolated tumor cells; MIC: micrometastases; MAC: macrometastases; RT: radiotherapy; CT chemotherapy; RCT: concomitant radiochemotherapy.
